# Financial challenges in Iran’s healthcare system arising from migrant populations: evidence from two hospitals in Yazd Province

**DOI:** 10.1186/s12913-025-13755-w

**Published:** 2026-01-10

**Authors:** Asgar Aghaei Hashjin, Mohammad Hossein Ghafoori, Mahdie Ghane, Banafshe Darvishi Teli, Mahsa Kaffashpour yazdi

**Affiliations:** 1 Department of Healthcare Services Management, School of Health Management & Information Sciences, Iran University of Medical Sciences, Tehran, Iran; 2 Department of Health Economics, School of Health Management and information Sciences, Iran University of Medical Sciences, Tehran, Iran; 3 Health policy and Management Research Center, Department of Health services Management, School of Public Health, Shahid Sadoughi University of Medical Sciences, Yazd, Iran; 4 Health Management and Economics Research Center, Iran University of Medical Sciences, Tehran, Iran; 5 Department of Management and Accounting, Faculty of Humanities and Arts, Technical and Vocational University (TVU), Tehran, Iran

**Keywords:** Health policy, Health insurance and reimbursement, Health economics, Immigrants, Refugees, Uncompensated cost, Economic burden

## Abstract

**Background:**

A review of the literature on the issues faced by foreign nationals in the area of health economics shows that researchers have mainly focused on the problems of immigrants and refugees in accessing health care services but the problems of the health system, particularly hospitals has been overlooked in this regard. The aim of this study is to investigate the uncompensated healthcare costs and the bed occupancy rates of the two main hospitals in Yazd province, due to immigrants.

**Methods:**

This descriptive and applied study was conducted in 2023 and 2024 in Yazd province, Iran. The study population included all documented inpatients of two hospitals affiliated to Shahid Sadoughi University of Medical Sciences, Shahid Rahnemoon and shahid Sadoughi. The sample size of the study was 338,886 hospitalized patient records. The data was extracted from the HIS system, and then categorized and analyzed using Excel 2019.

**Results:**

From 304,202 inpatients in two hospitals 34,425 (10%) were Afghani. Each patient had about 5926 dollars cost for health care system and 18.5% of costs were remain uncompensated. 77percents of patients were not under any health insurance coverage. The most used service was emergency services.

**Conclusion:**

Despite the existence of simple laws for Afghan insurance in Iran, they have little interest in getting insured, which has turned into a financial burden for the healthcare system in the country. Additionally, the large number of Afghan patients in public healthcare units has become a barrier to access affordable medical services for Iranian patients.

## Background

Large migrant and refugee populations can place considerable strain on healthcare systems worldwide by increasing demand for services, raising the need for specialized care, and generating uncompensated costs for hospitals [1–3]. Iran, as one of the largest host countries for migrants—with nearly 4.5 million Afghan nationals living legally or illegally—faces similar challenges [4, 5]. This substantial presence, together with the country’s economic and social difficulties, exerts significant pressure on the healthcare system, particularly public hospitals that serve uninsured or underinsured populations [6–10].

From the healthcare system perspective, two major challenges are evident: overcrowding of public hospitals and uncompensated healthcare costs. Uncompensated care refers to hospital services for which providers receive no payment from patients or insurers [11–13]. In Iran, studies have shown that foreign patients are among the leading contributors to unreimbursed hospital expenses. For example, Sohrabi et al. reported that nearly 17% of the uncompensated costs in hospitals affiliated with Iran University of Medical Sciences were attributable to foreign nationals [14]. These pressures manifest as elevated bed occupancy rates in critical hospital departments, substantial increases in unreimbursed treatment costs, and mounting constraints on financial and human resources—conditions that, if sustained, threaten the long-term sustainability and quality of healthcare services [12, 13, 15].

A review of the literature indicates that most studies on migrant health have focused on the challenges faced by immigrants themselves, particularly in accessing healthcare services [1, 2, 16–18]. In contrast, the systemic impact of immigrant populations on host country healthcare infrastructure—including hospital overcrowding, increased uncompensated costs, and pressure on financial and human resources—has received limited attention [13, 14, 18, 19]. This highlights a significant research gap: there is insufficient evidence on how immigrant populations affect hospital operations and resource allocation in Iran. Addressing this gap is crucial for informing policies that ensure equitable access to healthcare for migrants while maintaining the sustainability and efficiency of the healthcare system [20–22] .

Given the outlined challenges and the identified research gap, this study aims to examine the impact of immigrant populations on hospital operations in Yazd province, focusing on uncompensated healthcare costs and bed occupancy rates. By concentrating on systemic consequences rather than patient-level challenges, the research provides evidence-based insights to help policymakers manage hospital resources efficiently, ensuring both sustainability and equitable access to care [14, 21, 23]. Moreover, the study addresses the critical need to understand how hospitals adapt to the presence of a large migrant population and to identify strategies that can mitigate the financial and operational pressures associated with uncompensated care [14, 16, 24].

## Methods

### Study design

This descriptive cross-sectional and applied study was conducted in 2023–2024 in Yazd province, Iran. The study followed the guidelines of the EQUATOR Network.

### Study population and setting

The study population included all documented inpatients of two major governmental hospitals affiliated with Shahid Sadoughi University of Medical Sciences: Shahid Rahnemoon and Shahid Sadoughi Hospitals. These hospitals serve a large proportion of uninsured or underinsured patients, including Afghan and other foreign nationals.

### Sampling method and sample size

A census sampling method was used. A total of 338,886 hospitalization records were extracted from the hospitals’ Health Information Systems (HIS). The extracted data included patient nationality, total billing amount, discount rate, and admission department.

### Variables and operational definitions

Data were extracted from HIS using a SQL Server query and categorized in Excel 2019. The main study variables included:

Hospital bed occupancy by migrants and refugees: Calculated as the ratio of foreign inpatients (primarily Afghan nationals) in each hospital department to the total number of patients.

Uncompensated healthcare costs: Operationally defined as the portion of billed costs for which the hospital did not receive payment from patients or insurers. This included unpaid bills, insurance gaps, and hospital-provided discounts for patients unable to pay. The ratio of uncompensated costs for foreign patients to total patient billing costs was calculated.

Insurance coverage: Proportion of foreign patients with any form of health insurance.

### Statistical analysis

Descriptive statistics were used to summarize patient demographics, bed occupancy ratios, and uncompensated costs. Percentages, ratios, and per-capita costs were calculated for each hospital and department. Data analysis was conducted using Excel 2019.

## Results

Table 1 shows the general status of the patients in two hospitals individually and collectively. The results showed that about 10% of the patients in the two main government hospitals of Yazd province (34,425 patients) are refugees, which is a very high number and ratio.

**Table 1:** Total ratio of inpatients

Hospital	Afghani (Percent)	Iranian (Percent)	Iraqi (Percent)	Total
Shahid Rahnemoon	15,442 (11.65)	117,008 (88.34)	0	132,450
Shahid Sadoughi	18,983 (9.2)	187,194 (90.67)	259 (0.13)	206,436
Total	34,425 (10.15)	304,202 (89.76)	259 (0.09)	338,886

As can be seen in Table 2, the highest demand of Afghan patients in Shahid Rahnemoon hospital was for emergency department services, which of course is the same for Iranian patients. Also, orthopedics, dialysis, general surgery and emergency departments receive more Afghan patients than other departments, and a greater proportion of patients in the aforementioned departments are Afghan nationals. The departments of urology, internal medicine, CCU and ambulatory surgery also had the lowest number of Afghan patients.

**Table 2:** Shahid Rahnemoon’s inpatients based on department and nationality

Department	Afghani (Ratio of Total Afghani Percent)		Iranian (Ratio of Total Iranian percent)		Ratio of Afghani	Total
Emergency Services	10,523	(68.15)	74,371	(63.56)	12.40	84,894
Orthopedic	1019	(6.60)	4518	(3.86)	18.40	5537
Neurosurgery	795	(5.15)	6946	(5.94)	10.27	7741
General surgery	596	(3.86)	4176	(3.57)	12.49	4772
Urology	594	(3.85)	8588	(7.34)	6.47	9182
Others	589	(3.81)	7356	(6.29)	7.41	7945
Dialysis	496	(3.21)	2554	(2.18)	16.26	3050
Maxillofacial surgery	481	(3.11)	3753	(3.21)	11.36	4234
Internal medicine	172	(1.11)	2706	(2.31)	5.98	2878
Intensive Care Units	95	(0.62)	833	(0.71)	10.24	928
Outpatient surgury room	63	(0.41)	964	(0.82)	6.13	1027
C.C.U	11	(0.07)	151	(0.13)	6.79	162
V.I.P	8	(0.05)	92	(0.08)	8.00	100
Total	15,442		117,008		100	132,450

Considering that Shahid Sadoughi has more diverse and different specialties compared to Shahid Rahnemoon hospital, the pattern of service consumption by refugees is also different in this hospital. However, the most requested services are still from emergency units. In Shahid Sadoughi hospital, the departments related to maternity, women, infants and children, thalassemia and dialysis had the highest percentage of Afghan patients admitted (Table 3).

**Table 3:** Shahid Sadoughi’s inpatients based on department and nationality

Department	Afghani		Iranian		Iraqi		Ratio of Afghani	Total
Childbirth	592	(3.12)	1617	(0.86)	0	(0.00)	26.80	2209
Thalassemia	1156	(6.09)	3668	(1.96)	40	(15.44)	23.77	4864
Midwifery Triage	1042	(5.49)	3488	(1.86)	0	(0.00)	23.00	4530
PICU	67	(0.35)	286	(0.15)	1	(0.39)	18.93	354
N.I.C.U	405	(2.13)	1768	(0.94)	2	(0.77)	18.62	2175
Gynecology	1417	(7.46)	6493	(3.47)	6	(2.32)	17.90	7916
Dialysis	510	(2.69)	3029	(1.62)	6	(2.32)	14.39	3545
Pediatric Emergency	1127	(5.94)	7271	(3.88)	5	(1.93)	13.41	8403
Orthopedic	505	(2.66)	4152	(2.22)	3	(1.16)	10.84	4660
Pediatric	655	(3.45)	5425	(2.90)	6	(2.32)	10.76	6086
Emergency Surgery Room	3000	(15.80)	25,118	(13.42)	18	(6.95)	10.66	28,136
General Surgery	594	(3.13)	5300	(2.83)	7	(2.70)	10.07	5901
Pediatric Oncology	240	(1.26)	2146	(1.15)	1	(0.39)	10.05	2387
High risk childbirth	66	(0.35)	647	(0.35)	1	(0.39)	9.24	714
Others	1333	(7.02)	13,262	(7.08)	11	(4.25)	7.63	17,471
Outpatient surgery room	414	(2.18)	5283	(2.82)	7	(2.70)	7.26	5704
Eye Surgury	438	(2.31)	5835	(3.12)	8	(3.09)	6.97	6281
Emergency services	3380	(17.81)	49,372	(26.37)	62	(23.94)	6.40	52,814
Vascular and thoracic surgery	184	(0.97)	2754	(1.47)	17	(6.56)	6.23	2955
E.N.T	445	(2.34)	6947	(3.71)	6	(2.32)	6.02	7398
Infectious diseases	180	(0.95)	2908	(1.55)	5	(1.93)	5.82	3093
Bone marrow transplantation	59	(0.31)	1131	(0.60)	10	(3.86)	4.92	1200
Intensive Care Units for Adults	101	(0.53)	2098	(1.12)	8	(3.09)	4.58	2207
Internal Medicine	438	(2.31)	9712	(5.19)	13	(5.02)	4.31	10,163
Cardiovascular	184	(0.97)	4231	(2.26)	5	(1.93)	4.16	4420
Oncology	157	(0.83)	3622	(1.93)	2	(0.77)	4.15	3781
Early Detection of Cancer	37	(0.19)	874	(0.47)	0	(0.00)	4.06	911
Pancreatic and liver surgery	62	(0.33)	1562	(0.83)	2	(0.77)	3.81	1626

However, regarding the insurance coverage of Afghan patients, it can be seen that 77% of them are not covered by any insurance. 13% were covered by general insurance of the Iranian Health Insurance Organization and 7% were referred due to accidents and were covered by accident insurance. In total, only 33% of refugees were covered by insurance.

**Table 4:** Uncompensated cost of Afghanis total bill

Hospital	Total Cost Rials ($)		Uncompensated Rials ($)		Percent
Shahid Rahnemoon	568,291,556,762	(109,960)	163,193,377,474	(31,577)	28.72
Shahid Sadoughi	740,542,132,529	(143,289)	79,433,747,304	(15,370)	10.73
Total	1,308,833,689,291	(253,249)	242,627,124,778	(46,946)	18.54

**Fig. 1:**
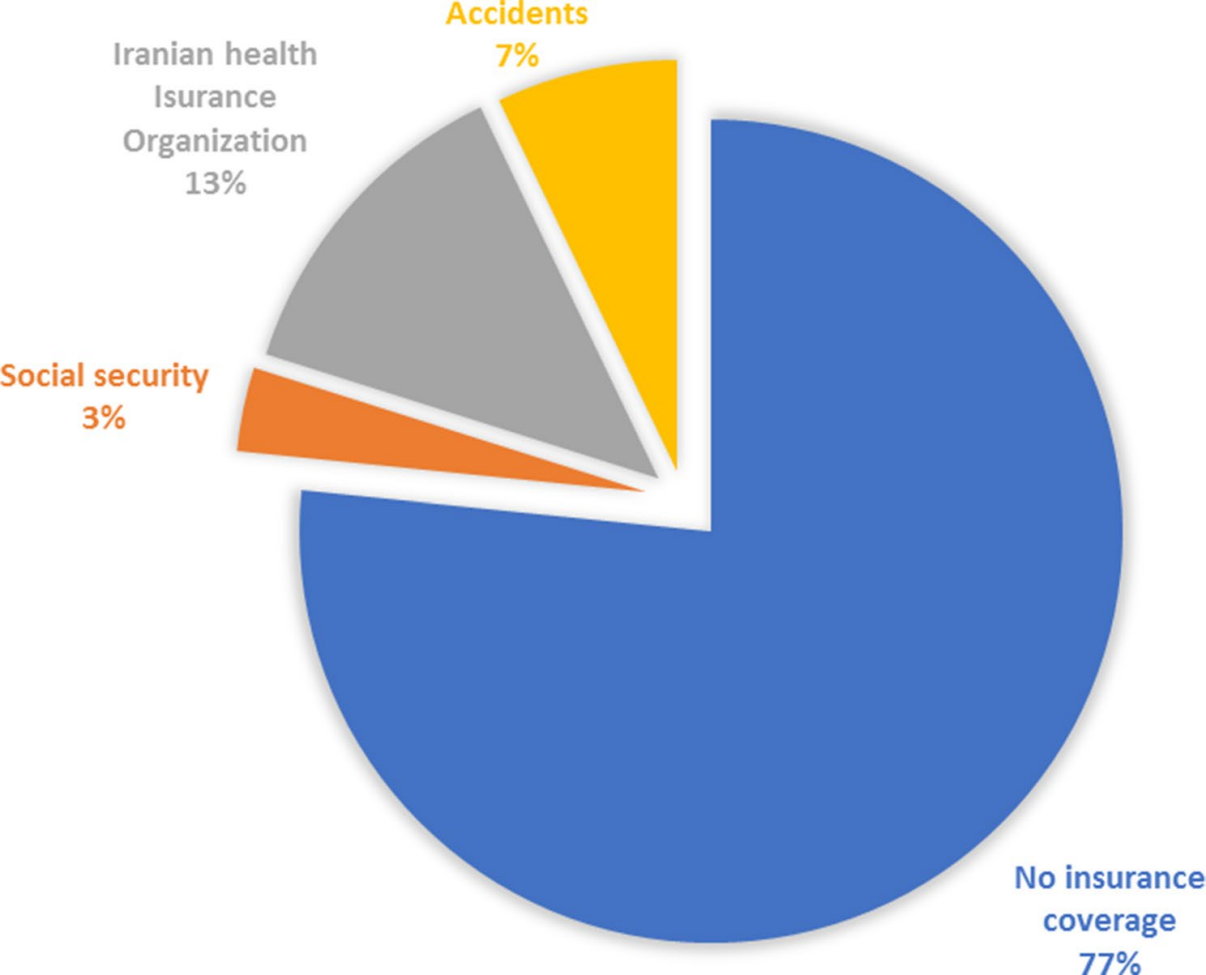
Insurance coverage of Afghani inpatients

The findings (Table 4) of the study indicated that about 18.5% of the total costs of Afghan patients remain uncompensated. This percentage is 28.7% for Shahid Rahnemoon and 10.7% for Shahid Sadoughi hospitals. The (Fig. 1) have been converted based on the exchange rate in Iran’s free market in 2024.

**Table 5:** The cost of each demanded service by Afghani

Department	*N* of inpatients	Total Cost ($)	$	Per capita ($)	$
Orthopedic	1,526	144,173,583,283	(278,964)	94,478,102	(183)
Neurosurgery	95	132,477,951,260	(256,334)	166,638,932	(322)
Emergency services	13,905	119,559,192,777	(231,338)	8,598,288	(17)
General Surgery	1,189	116,264,008,057	(224,962)	97,783,018	(89)
I.C.U for Adults	196	87,645,567,520	(169,587)	447,171,263	(865^*^)
N.I.C.U	405	84,426,331,037	(163,358)	208,460,077	(403^*^)
Gynecology	1,416	76,821,121,406	(148,643)	54,252,204	(105)
Internal medicines	610	60,481,907,513	(117,028)	99,150,668	(192)
Pediatric	655	44,099,464,364	(85,329)	67,327,427	(130)
Urology	545	43,738,901,480	(84,631)	80,254,865	(155)
Dialysis	1,006	41,210,145,370	(79,738)	40,964,359	(79)
Maxillofacial surgery	481	38,397,664,209	(74,296)	79,828,824	(154)
Cardiovascular	431	37,083,008,361	(71,753)	86,039,463	(166)
ENT	445	32,703,932,925	(63,280)	73,491,984	(142)
Pediatric Oncology	240	29,857,669,545	(57,772)	124,406,956	(241)
Oncology	157	24,185,778,320	(46,798)	154,049,543	(298)
Eye Surgury	438	23,720,078,316	(45,896)	54,155,430	(105)
Emergency Surgery Room	3,000	23,345,968,117	(45,173)	7,781,989	(15)
Childbirth	594	22,113,246,888	(42,787)	37,227,688	(72)
Vascular and thoracic surgery	184	19,557,202,253	(37,842)	106,289,143	(206)
Infectious diseases	180	18,176,465,696	(35,170)	100,980,365	(195)
PICU	67	15,265,469,213	(29,537)	227,842,824	(441)
C.C.U	113	13,983,386,547	(27,057)	123,746,784	(239)
Other Clinic Services	1,720	13,827,189,808	(26,755)	8,039,064	(16)
Pediatric Emergency	1,127	10,291,880,429	(19,914)	9,132,103	(18)
Neurology	93	6,421,502,992	(12,425)	69,048,419	(134)
Thalassemia	1,156	6,336,594,518	(12,261)	5,481,483	(11)
Bone marrow transplantation	59	5,907,228,705	(11,430)	100,122,520	(194)
Pancreatic and liver surgery	62	5,566,950,650	(10,772)	89,789,527	(174)
High Risk Childbirth	65	4,836,811,559	(9,359)	74,412,486	(144)
Outpatient surgery room	477	4,095,676,471	(7,925)	8,586,324	(17)
v.i.p	8	1,221,555,686	(2,364)	152,694,461	(295)
Midwifery Triage	1,042	867,738,671	(1,679)	832,763	(2)
Early Detection of Cancer	37	140,460,276	(272)	3,796,224	(7)
Total	34,424	1,308,801,634,222	(2,532,428)	3,062,855,569	(5,926)

Table 5 shows the total cost and cost per patient based on the type of services requested by patients of Afghan nationality. The most expensive services for healthcare system were orthopedics, neurosurgery, emergency services, general surgery, ICU and NICU. Also, the most expensive services in terms of cost per patient were neurosurgery, emergency and general surgery. The figures have been converted according to Iran’s free market exchange rate in 2024.

## Discussion

The findings of this study demonstrate that Afghan migrants utilize a considerable proportion of hospital services in Yazd province, particularly in maternity, pediatric, and intensive care departments. These results highlight two systemic challenges: (i) increased bed occupancy by migrants, which may reduce access for Iranian citizens, and (ii) significant uncompensated costs due to the high rate of uninsurance among Afghan patients. Importantly, while international literature often emphasizes structural and legal barriers that restrict migrants’ access to healthcare in host countries, our findings suggest that in Iran, the principal barrier is financial, stemming from lack of insurance coverage despite the existence of legal frameworks such as the Universal Public Health Insurance (UPHI) scheme.

Comparisons with international studies reveal both similarities and differences. Similar to findings in the United States and Europe, where uninsurance rates strongly predict uncompensated hospital care expenditures [6, 7, 25], our study confirms that lack of insurance among migrants is the primary driver of unreimbursed costs. However, unlike in many Western contexts where undocumented migrants face legal and administrative obstacles to access, Afghan patients in Iran are able to use a wide range of services, even in specialized hospital units. This paradox suggests that Iran’s inclusive policy environment, combined with weak enforcement of cost recovery mechanisms, enables access but simultaneously transfers the financial burden to hospitals.

The implications of these findings are significant. First, uncompensated care threatens hospital sustainability and may indirectly reduce quality of care. Second, the disproportionate use of critical departments such as maternity wards and NICUs by migrant patients creates resource competition, potentially undermining equity of access for Iranian citizens. Third, the lack of willingness to enroll in insurance schemes underscores the need for tailored strategies, including subsidies, community outreach, and enforcement mechanisms [5, 26, 27].

Future research should explore the behavioral and socioeconomic reasons behind migrants’ reluctance to purchase insurance, as well as the long-term consequences of uncompensated care for hospital operations. Comparative studies across provinces would also clarify the heterogeneity of migrant health system impacts within Iran.

## Strengths and limitations

This study benefits from a large sample size and the use of hospital information system (HIS) data, which enhances accuracy and reduces recall bias. Moreover, it is among the few studies in Iran to shift the focus from patient-level challenges to systemic consequences of migration on hospital operations. However, the study has several limitations. First, it was limited to two hospitals in Yazd province, which may not fully represent the situation across Iran. Second, the analysis was based on administrative records, and thus patient-level socioeconomic factors or reasons for lack of insurance could not be explored. Finally, the cross-sectional design precludes assessment of long-term financial trends and causality.

## Conclusion

This study demonstrates that Afghan migrants impose dual challenges on Iran’s healthcare system: high utilization of hospital services, particularly in critical departments, and a substantial burden of uncompensated care due to lack of insurance coverage. Unlike in many host countries where legal barriers limit migrant access, Iran’s inclusive policies have facilitated access but transferred the financial consequences to hospitals.

To address these challenges, actionable policy measures are needed. Hospitals should strengthen cost-recovery mechanisms such as prepayment or installment plans while safeguarding ethical obligations to provide emergency care. At the national level, expansion of subsidized insurance coverage and targeted international assistance through agencies such as UNHCR are critical to reducing uncompensated costs. International donors could directly support hospitals serving large migrant populations, thereby alleviating financial strain.

A notable difference was observed in the proportion of uncompensated costs between Shahid Rahnemoon and Shahid Sadoughi hospitals, with the rate being considerably higher in the former. This disparity can be explained by differences in cost recovery mechanisms and the nature of patient admissions. Shahid Sadoughi Hospital has established a dedicated unit responsible for managing and recovering payments from Afghan patients at the time of discharge. This unit actively pursues collection through Iranian guarantors linked to the patients, accepts postdated checks, arranges installment payments, and investigates patients’ financial capacity to facilitate reimbursement. Furthermore, this hospital applies stricter prepayment requirements for elective (non-emergency) cases. In contrast, Shahid Rahnemoon Hospital functions as the main trauma and emergency referral center in the province, where most admissions are unplanned and urgent. Under such circumstances, it is neither practical nor ethical to request prepayment or financial guarantees prior to providing care. As a result, a larger share of total treatment costs remains uncompensated at this hospital.

One limitation of this study is its focus on only two hospitals in Yazd province, and therefore the findings may not be fully representative of the situation across all Iranian provinces. Nevertheless, Shahid Sadoughi and Shahid Rahnemoon hospitals account for approximately half of the hospital beds in Yazd. The province also serves as a referral hub for the southeast of the country, including Kerman, Sistan and Baluchestan, and Bandar Abbas, as well as a transit point for Afghan patients traveling to northern provinces and the capital. Thus, examining these two hospitals can provide valuable insights into the healthcare utilization behaviors of Afghan migrants.

Furthermore, the cultural and social characteristics of the Afghan community are relevant to interpreting these findings. Fertility practices among Afghan populations are largely unrestricted by age, and in some groups, certain forms of contraceptive use are considered religiously impermissible. Therefore, just as the findings cannot be directly generalized nationally, it is also difficult to assume significant regional variation in fertility-related behaviors among Afghan communities. Consequently, any extrapolation of these results to a national level should be undertaken with caution, taking into account demographic and cultural factors.

Based on field observations and informal discussions with hospital staff, several potential explanations can be proposed for the high proportion of uninsured Afghan patients. First, financial constraints appear to be a major barrier—many Afghan migrants are either unable or unwilling to pay the insurance premium due to unstable income and competing living costs. Second, there seems to be a cultural component: insurance is often perceived as unnecessary until illness occurs, reflecting limited awareness of the preventive and protective value of health insurance. Third, a considerable number of Afghan residents have irregular or undocumented legal status, making them ineligible for official insurance enrollment. In some cases, fear of identification or legal consequences discourages them from registering for insurance, even when technically eligible. These factors together may explain the persistently high uninsured rate observed in our data. Future qualitative research—such as in-depth interviews with Afghan residents and health administrators—is warranted to validate these hypotheses and to inform targeted policy interventions aimed at increasing insurance coverage among this vulnerable group.

## Data Availability

The datasets used and/or analysed during the current study are available from the corresponding author on reasonable request.

## Abbreviations

HIS: Health Information System
PICU: Pediatric Intensive Care Units
NICU: Neonatal Intensive Care Units
IHIO: Iran Health Insurance Organization
UPHI: Universal Public Health Insurance

## References

[1] Hosseini Divkolaye NS, Burkle FM Jr. The enduring health challenges of Afghan immigrants and refugees in Iran: a systematic review. PLoS Curr. 2017;9. 10.1371/currents.dis.449b4c549951e359363a90a7f4cf8fc4 PMC5554007 28856065

[2] Roozbeh N, Sanati A, Abdi F. Afghan refugees and immigrants health status in iran: A systematic review. Population. 2018;3:4.

[3] Grimm JW, Wells JL. Illegal immigrants in the emergency department: an ethical dilemma for nurses? J Emerg Nurs. 2009;35(2):127–8. 19285176 10.1016/j.jen.2008.08.018

[4] Citaristi I. *United nations high commissioner for refugees—UNHCR*, in *The Europa directory of international organizations 2022*. Routledge; 2022. pp. 220–40.

[5] Mohammadi Y, Khodaverdi H, Siraki GK. CHALLENGES AND OPPORTUNITIES OF AFGHANISTAN NATIONALS’PRESENCE IN THE ISLAMIC REPUBLIC OF IRAN AND ITS IMPACT ON THE NATIONAL SECURITY DURING TALIBAN’S TENURE AND POST-TALIBAN GOVERNANCE. European Journal of Political Science Studies; 2018.

[6] Divkolaye N, Burkle F Jr. The enduring health challenges of Afghan immigrants and refugees in Iran: a systematic review. PLoS Curr 2017;9. 10.1371/currents.dis.449b4c549951e359363a90a7f4cf8fc4 PMC5554007 28856065

[7] McGrath J. Overcharging the uninsured in hospitals: shifting a greater share of uncompensated medical care costs to the federal government. QLR. 2007;26:173.

[8] Nadjarzadeh A, et al. Determining the content and needs assessment a mobile-based self-care program in infertile men. BMC Med Inf Decis Mak. 2023;23(1):258. 10.1186/s12911-023-02366-2 PMC10644630 37957627

[9] Sumra KB, et al. The refugees and health crisis: migration policy management and government response to Afghan migrants. BMC Health Serv Res. 2025;25(1):218. 39920634 10.1186/s12913-025-12290-y PMC11806718

[10] Abasi A, Nazari A, Moezy A, Fatemi Aghda SA. Machine learning models for reinjury risk prediction using cardiopulmonary exercise testing (CPET) data: optimizing athlete recovery. BioData Mining. 2025;18(1):1-25. 10.1186/s13040-025-00431-2 PMC11834553 39962522

[11] Winters M, et al. A systematic review on the use of healthcare services by undocumented migrants in Europe. BMC Health Serv Res. 2018;18(1):30. 29347933 10.1186/s12913-018-2838-y PMC5774156

[12] Ornelas IJ, Yamanis TJ, Ruiz RA. The health of undocumented Latinx immigrants: what we know and future directions. Annu Rev Public Health. 2020;41(1):289–308. 32237989 10.1146/annurev-publhealth-040119-094211 PMC9246400

[13] Gooshki ES, Rezaei R, Wild V. Migrants’ health in Iran from the perspective of social justice: a systematic literature review. Archives Iran Med (AIM). 2016;19(10). 27743440

[14] Sohrabi Anbouhi Z, et al. Factors affecting the uncompensated costs in hospitals of Iran university of medical sciences: panel data approach. J Health Adm. 2021;24(1):43–53.

[15] Langarizadeh M, et al. Identifying and validating the educational needs to develop a Celiac Self-Care system. BMC Prim Care. 2023;24(1):121. 10.1186/s12875-023-02076-8. PubMed PMID: 37316859. 37316859 10.1186/s12875-023-02076-8 PMC10265559

[16] Chen J, et al. Latino population growth and hospital uncompensated care in California. Am J Public Health. 2015;105(8):1710–7. 26066960 10.2105/AJPH.2015.302583 PMC4504330

[17] Green BA, Latifi Y. No one smiles at me: the double displacement of Iranian migrant men as refugees who use drugs in Australia. Social Sci. 2021;10(3):85.

[18] Abasi A, et al. An investigation into telemedicine utilization for refugee mental health: a systematic review. Globalization Health. 2025;21(1):31. 40420111 10.1186/s12992-025-01119-2 PMC12105209

[19] Karami M, et al. Enhance hospital performance from intellectual capital to business intelligence. Radiol Manage. 2013;35(6):30–5. 24475528

[20] Ghiasi K, et al. Strategies for improving migrant health in iran: a realist review. Globalization Health. 2025;21(1):42. 40731414 10.1186/s12992-025-01133-4 PMC12308935

[21] Kiani MM et al. Refugees and sustainable health development in Iran. Archives Iran Med (AIM). 2021;24(1). 10.34172/aim.2021.05 33588565

[22] Langarizadeh M, et al. The nutritional content required to design an educational application for infertile women. BMC Womens Health. 2023;23(1):22. 36650480 10.1186/s12905-023-02156-y PMC9843656

[23] Amuzadeh-Araei S, Takian A, Jabbari A. Universal public health insurance for Afghan refugees in iran: a contextual analysis. Global Health. 2025;21(1):46. 40796851 10.1186/s12992-025-01143-2 PMC12345101

[24] Moezzi SMI, et al. Barriers and facilitators to primary healthcare utilization among immigrants and refugees of low and middle-income countries: a scoping review. Globalization Health. 2024;20(1):75. 39449084 10.1186/s12992-024-01079-z PMC11515291

[25] van der Boor CF, et al. Systematic review of factors associated with quality of life of asylum seekers and refugees in high-income countries. Confl Health. 2020;14(1):48. 32699551 10.1186/s13031-020-00292-y PMC7370437

[26] Etemadi M, et al. Identifying challenges and enabling factors in utilizing health care services by undocumented immigrant women: a scoping review of qualitative research. J Health Popul Nutr. 2025;44(1):301. 40836266 10.1186/s41043-025-01051-6 PMC12369077

[27] Pérez-Urdiales I. Undocumented immigrants’ and immigrant women’s access to healthcare services in the Basque country (Spain). Global Health Action. 2021;14(1):1896659. 33975531 10.1080/16549716.2021.1896659 PMC8118419

